# Supplementary material for: Ferroptosis-Mediated Cell Death Induced by NCX4040, The Non-Steroidal Nitric Oxide Donor, in Human Colorectal Cancer Cells: Implications in Therapy
Source: Cells. 2023 Jun 14;12(12):1626. doi: 10.3390/cells12121626 (PMC10297642; doi:10.3390/cells12121626)
Supplement: Supplementary file 1 [file cells-12-01626-s001.zip › cells-2432235-supplementary.pdf]

Name	Molecular Formula	Annotated Feature (Index|m/z|RT)	Species	Measured *m/z*	Theoretical *m/z*	Absolute Mass error (ppm)
Glutathione	$C_{10}H_{17}N_3O_6S$	373|308.091|0.668	[M+H]+	308.091	308.0911	0.32
Glutathione disulfide	$C_{20}H_{32}N_6O_{12}S_2$	161|613.15896|0.905	[M+H]+	613.159	613.1592	0.33
Taurine	$C_2H_7NO_3S$	525|124.00746|0.488	[M-H]-	124.0075	124.0074	0.81
Arachidonic acid	$C_{20}H_{32}O_2$	147|303.23339|8.722	[M-H]-	303.2334	303.233	1.32
Acetylcarnitine	C9H14NO4	317|204.12291|1.148	[M+H]+	204.1229	204.123	0.49
Propionylcarnitine	C10H20NO4	125|218.13863|3.42	[M+H]+	218.1386	218.1387	0.46
(Iso)butyrylcarnitine	C11H21NO4	325|232.15419|3.806	[M+H]+	232.1542	232.1543	0.43
Succinylcarnitine	C11H19NO6	207|262.12845|1.402	[M+H]+	262.1285	262.1285	0
Lauroylcarnitine	C19H37NO4	298|344.27961|7.506	[M+H]+	344.2796	344.2795	0.29
2-Tetradecenoylcarnitine	C21H39NO4	24|370.29523|7.804	[M+H]+	370.2952	370.2952	0
Palmitoylcarnitine	C23H45NO4	154|400.34219|8.96	[M+H]+	400.3422	400.3421	0.25
Stearoylcarnitine	C25H49NO4	270|428.37346|9.724	[M+H]+	428.3735	428.3734	0.23
Adenosine-5'-triphosphate	C10H16N5O13P3	508|508.00271|0.583	[M+H]+	508.0027	508.003	0.59
Nicotinamide adenine dinucleotide (NAD)	C21H28N7O14P2	211|664.11647|1.878	[M]+	664.1165	664.1164	0.15
Flavin adenine dinucleotide (FAD)	C27H33N9O15P2	392|786.16404|4.075	[M+H]+	786.164	786.1644	0.51
Guanosine-5'-triphosphate	C10H16N5O14P3	346|523.99805|0.583	[M+H]+	523.9981	523.9979	0.38
Adenosylmethionine	C15H22N6O5S	109|399.14424|0.584	[M+H]+	399.1442	399.1445	0.75
Methyladenosine	C11H15N5O4	753|282.11966|1.029	[M+H]+	282.1197	282.1197	0

Measured Retention Time (min)	Reference Retention Time (min)	Retention Time Difference (s)	MS/MS
0.67	0.77	-6	yes
0.91	-	-	yes
0.49	0.45	2.4	yes
8.72	-	-	no
1.15	1.06	5.4	yes
3.42	3.4	1.2	yes
3.81	3.8	0.6	yes
1.4	1.64	-14.4	yes
7.51	7.51	0	yes
7.8	8.1	-18	yes
8.96	9.05	-5.4	yes
9.72	9.92	-12	yes
0.58	0.7	-7.2	yes
1.88	1.72	9.6	yes
4.08	3.96	7.2	yes
0.58	0.57	0.6	yes
0.58	0.66	-4.8	yes
1.03	-	-	yes
